# Efficacy and patient acceptability of the continence dish

**DOI:** 10.1007/s00192-021-04969-7

**Published:** 2021-09-14

**Authors:** Kate H. Moore, Wendy Allen, Katrina Parkin, Fiona Beaupeurt, Chris Chan, Zhuoran Chen

**Affiliations:** The Pelvic Floor Unit, Department of Urogynaecology, University of New South Wales at St. George Hospital, Gray Street, Kogarah, Sydney, NSW 2217 Australia

**Keywords:** Continence dish, Stress incontinence, Vaginal device

## Abstract

**Introduction and hypothesis:**

The continence dish has been a treatment option since 2002 for women with stress urinary incontinence (SUI) who decline surgery, but few quantitative objective efficacy data are published. We aimed to determine the efficacy and acceptability of this device for pure SUI or mixed incontinence (MUI).

**Methods:**

Prospective interventional cohort study of 100 women with SUI or stress-predominant MUI who were interested to use the device; International Consultation on Incontinence Questionnaire (ICIQ) was primary outcome measure; 24-h pad test and Incontinence Impact Questionnaire (IIQ) were secondary outcomes. Acceptability was determined by device retention for 4 weeks, adverse events and ability to self-insert the device.

**Results:**

Of 100 suitable women, 9 were not actually fitted, and 27 did not complete (acceptability: 64/100). The rate of adverse events was 7.7%, with 62.5% of users able to self-insert the device: 22 (34%) had pure SUI; 66% had MUI. In SUI, 68% were ‘dry’ on ICIQ median value 4.0 (IQR 2.5–8.5); 88% were dry on 24-h pad test (median 0.0, IQR 0.0–8.5). The “dry rate” was lower in MUI: 36% for ICIQ (median 9.0, IQR 5.0–15.0) and 62% for 24-h pad test (median 6.2, IQR 0.95–19.7). A “good” response on IIQ occurred in 88% of SUI and 69% of MUI.

**Conclusion:**

These new data showing strong objective benefits of the continence dish should be further validated by randomized trials, but this information should be made available to women seeking treatment options for SUI/MUI (particularly in view of concerns regarding mesh mid-urethral slings).

## Introduction

The management of stress urinary incontinence (SUI) traditionally comprises pelvic floor physiotherapy and/or surgery. Nevertheless, some women fail to respond to conservative therapy but do not wish to consider surgery; younger women who are incontinent during certain activities may wish to delay surgery until completion of their families, or older women may be concerned by the surgical risks of a non-life-threatening condition [1].

In the last 3 decades, various vaginal devices have been developed to treat women with stress incontinence that decline surgery [2–6]. The Introl Bladder Neck Support Device provides continence to 62% of women with SUI [5] who may also have prolapse, while the Contiform, shaped like a hollow tampon and with a proven dry rate of 54% on pad test [6], is not suitable for women with uterovaginal prolapse.

**Fig. 1:**
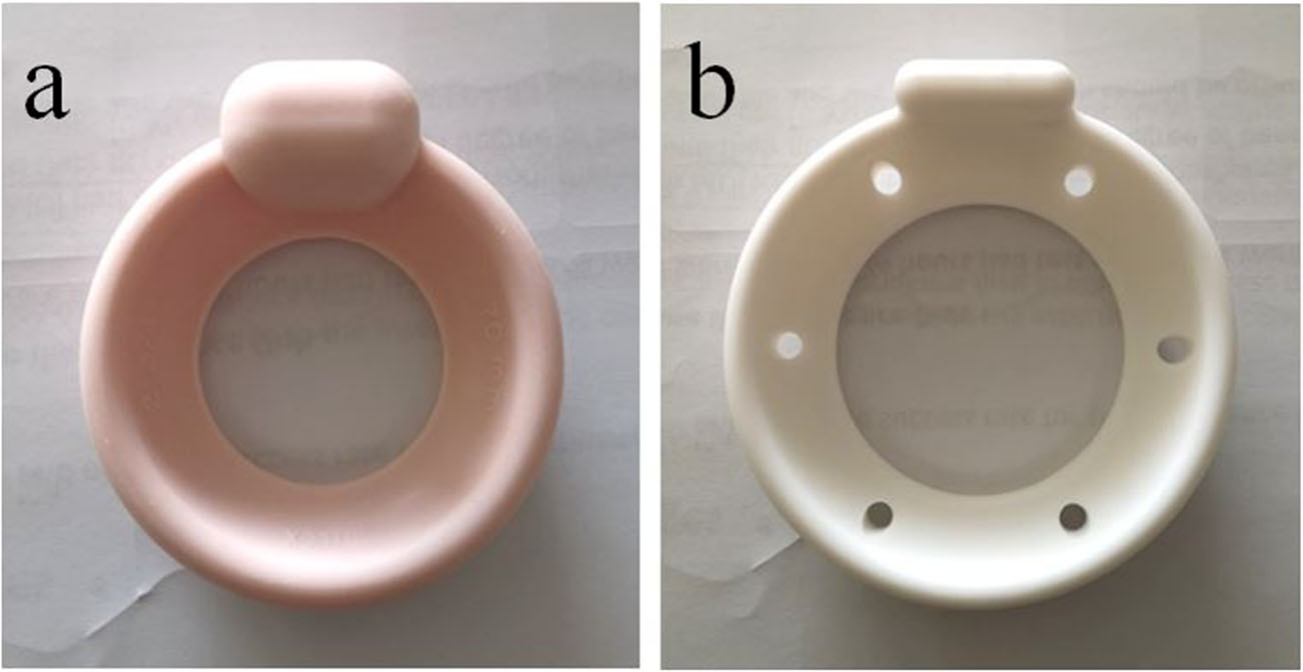
**a** Pink continence dish, 65 mm in diameter. **b** White continence dish, with perforations to allow mucous drainage/ mucosal seal

**Fig. 2:**
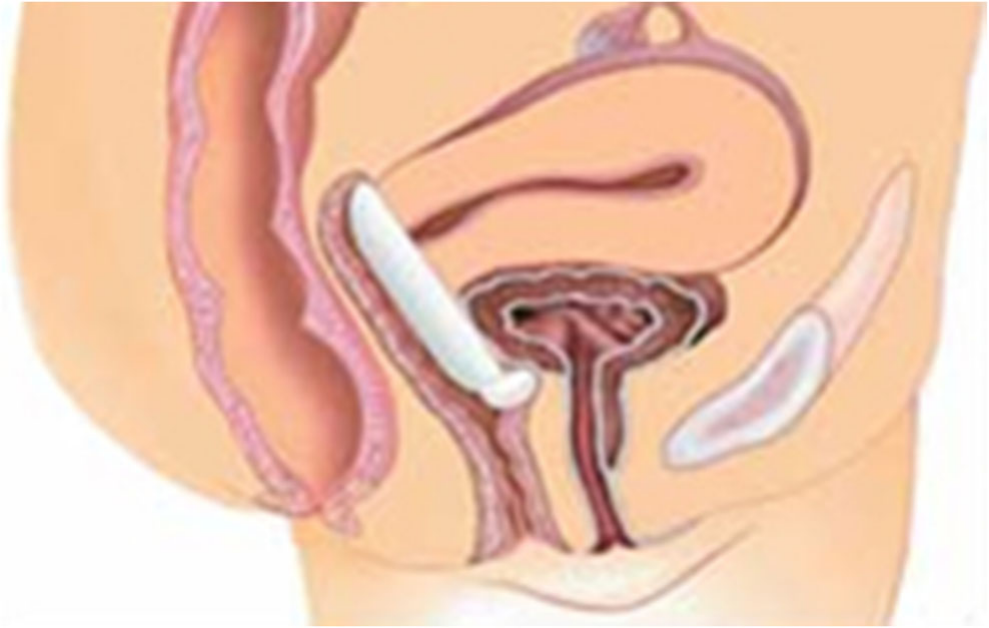
Schematic diagram of continence dish sitting correctly with protuberance at bladder neck

A further new device, the continence dish, has been in use since 2002. It is shaped in the form of a small round “dish” with a hollow centre and a central protuberance (Fig. 1a) and is positioned to sit underneath the urethra, creating a support in a similar fashion to the suburethral sling (Fig. 2). Because of its basic “ring” configuration, it can also be used to support pelvic organ prolapse. There are two variable forms of this device, which come in eight different sizes; a pink device with a larger protuberance for more urethral support (Fig. 1a) and a white ring with perforations in the membrane to allow mucous to drain, thereby creating a “mucosal seal” (Fig. 1b). However, although there have been two review articles on the continence dish [7, 8] and a randomized controlled trial versus pelvic floor physiotherapy [9, 10], which employed Patient Global Impression of Improvement (PGII) as the primary outcome, objective outcome data for this device remain quite limited.

We aimed to accurately measure the efficacy of this device in women with pure stress and mixed incontinence, using internationally recognized quantitative outcomes, and to measure patient acceptance, usability and adverse events. We hypothesised that the device would be more effective for women with stress incontinence, but should also benefit those with mixed incontinence (because elevation of the urethrovesical junction may render the urge component easier to treat).

## Materials and methods

This was a prospective interventional cohort study of 100 patients with pure stress incontinence and stress-predominant mixed incontinence, attending a regional referral Urogynaecology Unit in a large metropolitan hospital between 2015 and 2019. A detailed history was collected from all patients at the first visit, with a physical examination that included POP-Q and training in contraction of the pelvic floor muscle, with urinalysis. Baseline demographics, ICIQ and a 3-day bladder chart were collected. All patients were advised to perform pelvic floor muscle training at this visit.

At the second visit, patients were invited to join the audit study of the dish among other options, including pelvic floor physiotherapy or surgery. They were made aware they were under no obligation to continue with the study should they change their mind or opt for an alternative treatment. Patients were not formally “selected” to enrol (except that women with pure urge incontinence were not invited to participate as the device is designed to treat stress incontinence). Pure stress incontinence was defined as women only complaining of leakage with cough or exertion, and fewer than eight voids per 24 h, with normal voided volumes (300–400 ml) on bladder diary. Stress-predominant mixed urinary incontinence (MUI) was defined as women with a main complaint of stress incontinence, but occasional episodes of urge incontinence and smaller bladder volumes on bladder diary. Women with mixed stress and urge incontinence symptoms were offered the choice to try the device before receiving anticholinergic medications, which they purchased from pharmacies in the normal manner. Women were free to continue performing pelvic floor muscle training, but formal pelvic physiotherapy including digital assessment was problematic with the device in situ.

Women were given an illustrated information sheet about the fitting and usage of the continence dish and invited to return within 2–14 days for fitting with a specialist nurse. Patients who declined to join/continue at various stages of the study were noted. Because the device has been used since 2006 in this department and others internationally, yet minimal objective outcome data could be found in the literature, the approval of the local South Eastern Sydney Local Health District Ethical Committee (Ref 17/26) (IRB) was sought on the basis that this study was an audit of a clinical treatment.

### Fitting of the device

All patients at the unit who have a continence dish fitted have a separate session with an experienced nurse continence advisor (NCA). Each patient was invited to ask questions about the information sheet prior to fitting the device. Based on the assessment of the length and width of the vaginal canal, the NCA fitted an appropriately sized continence dish. If this was unsatisfactory, then either a smaller or larger size was fitted as appropriate. The size was adjusted to achieve a snug but comfortable fit within the vagina, with the round protuberance sitting underneath the urethra (as per Fig. 2). Immediately after, the patient was requested to cough to ensure no extrusion of the device. The patient then left the unit for a short period to ensure that they remained comfortable with the continence dish in situ, as a simulation of their usual activities of daily living. During this period away, patients were requested not to void and encouraged to have oral fluids to assess the immediate efficacy of the continence dish with fluid in the bladder. Upon returning, the patient was assessed for any leakage during cough with a full bladder and their ability to void with continence dish in situ. Patients returned to the unit after 2 weeks for a follow-up visit with the NCA. During this visit, information regarding suitability of the device was collected from the patient, including comfort, ability to defecate and void, and any adverse effects. In addition, patients were also encouraged to learn the self-insertion and removal of the device.

### Assessment of acceptability and efficacy

Acceptability was assessed by the continued usage of the continence dish for 4 weeks and beyond, and any adverse effects were reported by patients.

Efficacy was assessed by a pre- and post-treatment International Consultation on Incontinence Questionnaire (ICIQ), which comprises four questions with a total score of 21. An ICIQ score ≤ 6 is reported as success in postoperative surgical trials [11]. Objective efficacy was measured by a post-treatment 24-h pad test with dish in situ. The 24-h pad test [12] is a well-established method to assess the urine loss of women in their natural environment. Patients were given five pre-weighed pads, sealed in a zip lock bag and measured to an accuracy of 0.1 g on an electronic balance. To prevent undue evaporation, patients were requested to seal the pads in the zip lock bag and return them in a provided envelope within 7 days. Upon return, the pads are weighed using the same electronic balance to assess the urine loss over the 24-h period. “Dry” is traditionally defined as < 11.5 g urine loss per 24 h [12], although a more strict definition is also published [13].

The short form of the Incontinence Impact Questionnaire (IIQ-7) is a measure of efficacy and quality of life. This questionnaire comprises seven questions with a total score of 21, but is converted to a scale of 0 to 100%, with a “good” response defined as < 50%, “moderate” as 50–70% and “poor” as > 70% [14, 15]. A patient satisfaction score was administered, consisting of three visual analogue scales regarding (1) satisfaction with the continence dish (score of 10), (2) satisfaction with overall treatment for incontinence (score of 10) and (3) a rating for the change in bladder control since using the continence dish (score of 5, total score = 25). The raw score × 4 yields a percentage score.

Patients were analysed as a total cohort, with subgroup analysis performed separately for the SUI and MUI. Because this was an audit study, formal sample size was not calculated, but numbers of patients were similar to or greater than in previous studies [8–10, 17–19]. All descriptive data are quoted as median and interquartile range, as the data are not normally distributed. Statistical analysis was performed by Wilcoxon signed rank for all pre- and post-treatment analyses and by Mann-Whitney test for subgroup comparisons, using SPSS version 25.0.

## Results

### Patient acceptability

There were 100 consecutive patients with stress predominant incontinence who expressed interest in being fitted with the continence dish and were invited to participate in this audit. The median age (IQR) of all participants was 66.5 (49–76) years. Of these, nine women were not actually fitted with the device. Having read the information sheet, they realized that the device would not suit their lifestyle (*n* = 2), they previously always extruded tampons (*n* = 3), they had become medically unwell (*n* = 2), they were reluctant to self-insert but were sexually active (*n* = 1), and one never returned to the unit.

Of the remaining 91 patients, 7.7% (7/91) reported adverse effects: 3.3% reported pain while using the continence dish, 2.2% reported vaginal bleeding, and 1.1% reported erosion and thrush. Table 1 shows that 6 of these adverse events occurred in the 27 women who did not continue to use the device, but 1 of the 64 continuing participants also had minor episodes of vaginal erosions with dark brown vaginal staining, which responded quickly to removal of the ring with salt baths and daily Ovestin for 2 weeks.

**Table 1:** Reasons for non-continuations

Adverse events: 6/91 (6.6%)	
Pain/discomfort with device in situ	3
Vaginal bleeding/staining	2 (+1 in a continuing participant)
Recurrent vaginal thrush and erosion	1
Other reasons: 27/91 (29.7%)	
Unable to retain device	9 (+1 thrush erosion)
Minimal benefit	9
Chose to have surgery	6
Unable to remove device	1
Medically unfit to continue	1
Stopped sports, no more leak	1

Twenty-seven of 91 women (30%) did not continue to use the device, with the primary reasons stated as the inability to retain the device (9/27, 33%) and experiencing minimal benefit (33%), as shown in Table 1. This resulted in 64/91 (69%) subjects who continued participation in use of the continence dish. The median age of the 36 who could not be fitted or did not continue (66.5, IQR 49–76) did not differ from the age of the successful participants (median 66, IQR 48–75; Mann-Whitney *p* = 0.052). The median size of the successfully fitted continence dish was 65 (IQR 60–71.5) mm, and the median size for the women with pure stress incontinence or mixed incontinence was the same.

Forty of 64 women were able to self-insert and self-remove the device (62.5%). Of the participants, 22/64 (34%) had pure stress incontinence while 66% (42/64) had stress-predominant mixed incontinence. Baseline demographic data for the 64 participants are shown in Table 2.

**Table 2:** Baseline demographic data of completed patients

Patient characteristics (*n* = 64)	
Median age (years)	67 (IQR 50–76)
Postmenopausal	45/64 (70%)
Vaginal oestrogen use	42/64 (65%)
Pelvic organ prolapse	35/64 (55%)
Self-insertion/removal	40/64 (62.5%)
Type of incontinence	
Pure stress	22/64 (34%)
Mixed	42/64 (66%)

Of the 42 women with stress-predominant mixed incontinence, 15 (36%) chose not to use anticholinergic medications. Of the remaining 27, the anticholinergics used were Oxytrol patches (*n* = 10), Imipramine (*n* = 6), Vesicare (*n* = 3) and two cases each for Enablex, rapid release Oxybutynin and Mirabegron.

### Efficacy

**Fig. 3:**
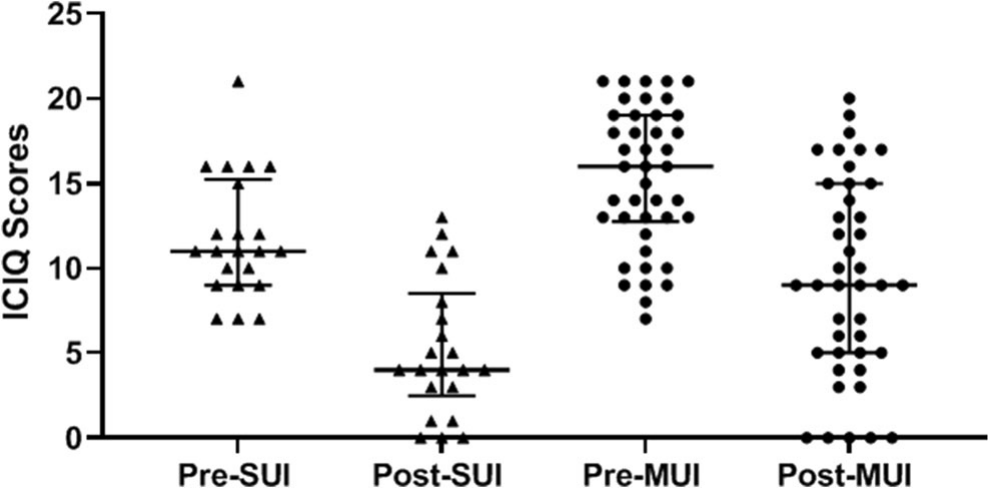
Results of ICIQ scores pre- and post-therapy, in relation to stress urinary incontinence (SUI) and mixed urinary incontinence (MUI). Data are presented as median ± IQR

As shown in Table 3, 68% of patients with stress incontinence were “cured’ on the ICIQ, and their scores were significantly reduced from a median of 11 pre-treatment to 4 post-treatment. A significant reduction in ICIQ also occurred in the mixed group (from 16 to 9), but only 38% were ‘cured’ on this outcome. The overall cure rate was 47% as a result (see Fig. 3). Subset analyses of patients with and without pelvic organ prolapse (POP) are shown.

**Table 3:** Continence dish outcome measure of completed patients

Outcome measure (*n*)	Pre-therapy (median ± IQR)	Post-therapy (median ± IQR)	Significance (*P* value)	% Patients dry or “good” while using device
ICIQ				
All (64)	14 (10.3–18)	7 (4.0–12.0)	< 0.0001	18/64 (28%)
Pure stress (22)	11 (9.0–15.3)	4 (2.5–8.5)	< 0.0001	12/22 (54%)
Mixed (42)	16 (12.8–19.0)	9 (5.0–15.0)	< 0.0001	6/42 (14%)
24-h pad test				
All (51)	–	4.5 (0.0–14.6)	–	34/51 (66%) (ITT 53%)
Pure stress (17)	–	0.0 (0.0–8.48)	0.041	15/17 (88%) (ITT 68%)
Mixed (34)	–	6.2 (0.95–19.7)		19/34 (56%) (ITT 45%)
IIQ < 50				
All (52)	–	16.7 (4.7–47.5)	–	65%
Pure stress (17)	–	14.3 (2.4–33.3)	0.394	76%
Mixed (35)	–	19.0 (4.7–61.8)		60%
Patient satisfaction score > 85%				
All (55)	–	19.0 (15–23)	–	42%
Pure stress (19)	–	21.0 (17.23)	0.098	53%
Mixed (36)	–	18.5 (15–22.8)		36%

Where: ICIQ, International Consultation on Incontinence Questionnaire; IIQ, Incontinence Impact Questionnaire; ITT, Intention to Treat

Significance values denoted for the ICIQ outcome measure present pre- and post-therapy comparisons for the given patient group, using Wilcoxon matched-pairs signed rank test. All other reported *p*-values represent the significance between pure stress and mixed patient groups for the given outcome measure, using Mann-Whitney test

Regarding the 24-h home pad test, 88% of women with stress incontinence who performed this test were dry, with a median loss of 0.0 g (IQR 0.0–8.45). However, five women did not complete it (three had ‘cured’ ICIQ scores of 1–4, while the fourth and fifth women had ICIQ scores of 5 and 7, respectively). Thus, the dry rate calculated on intention-to-treat (ITT) basis was 68% for stress incontinence. In the 42 women with mixed incontinence, pad tests were completed by 34 women, of whom 62% were dry [but on ITT analysis, dry rate was 50% with a median urine loss over 24 h of 6.2 g (IQR 0.95–19.7)]. The results for stress incontinent women were significantly better than those for mixed incontinence (*p* = 0.041).

Table 3 also indicates changes in the quality of life on IIQ. Overall, 75% of patients reported “good” quality of life on the IIQ. Consistent with the objective efficacy outcome measures, women with pure stress incontinence accounted for a larger proportion; 88% reported “good” quality of life compared to 69% of women with mixed incontinence (difference between groups not significant, *p* = 0.39).

Regarding the visual analogue patient satisfaction score, 53% of women with stress incontinence self-rated their satisfaction > 84%, as opposed to 33% of women with mixed incontinence.

## Discussion

It was surprising to find that despite several publications regarding successful fitting of continence pessaries, only one small previous report gave objective data (e.g., pad test or ICIQ) regarding the reduction of incontinence achieved by such a device. For example, of 38 women with SUI fitted with an “incontinence ring pessary with diaphragm” (Milex) [16], only 6 (16%) wished to continue the device after 12 months. The results of their “semiquantitative pad test” diminished from a baseline mean of 11 g to 1.2 g at 12 months, with daily leaks falling from 4.1 to 1.5 per day on 7-day diary and IIQ falling from 17 to 2.

A retrospective cohort study [8] of 119 women in 2004 showed successful fitting of a variety of continence dishes in 89% of cases and continued use occurred in 45% at 6 months, but no objective outcome data were provided. In 2008, the mechanism of action of incontinence pessaries was studied: Komesu et al. [17] found a significant increase in the function urethral length and significant elevation of the bladder neck, with a reduced rate of bladder neck funnelling on MRI in 15 women. Noblett et al. (2008) showed a significant increase in maximum urethral closure pressure in 33 women with an incontinence dish pessary in situ [18].

In 2010, two reports [9, 10] of a randomized controlled trial of either an incontinence ring or an incontinence dish versus behavioural training (pelvic floor muscle strengthening with bladder training) or combination therapy were published, the “ATLAS” trial. The main outcome measures were Patient Global Impression of Improvement and the stress incontinence subscale of the Pelvic Floor Distress Inventory [9]. Results showed that 40% of the continence pessary patients were “much better” or “very much better” on PGII, and 33% had no bothersome stress incontinence symptoms on the PFDI. On bladder diary, 46% of women had > 75% reduction in weekly incontinence episodes. A further report of this RCT [10] showed that 92% of women were fitted successfully with an incontinence ring (*n* = 122) or a dish (*n* = 113) out of 266 recruits and that neither vaginal length nor genital hiatus predicted successful fitting, but objective measures of incontinence were not shown.

Unlike the Contiform, the continence dish doubles as a pessary for pelvic organ prolapse (POP). As seen in Table 2, more than half (35/60) of the women in the current study had concomitant POP; however, Table 3 indicates that the results were very similar for those with SUI, but the efficacy was significantly better in the mixed group who had no prolapse. In addition, the continence dish does not require daily removal unlike the Contiform (which requires daily removal and lasts for only 45 insertions) and therefore involves substantially lower financial commitment.

As demonstrated, the continence dish is effective for 68% of patients with pure stress incontinence. Note that we used the definition of “dry” for the pad test employed by Ulmsten et al. [19] to evaluate the success of the TVT operation (so that women can compare the same test of “cure”). Using a more stringent published pad test definition of “dry” (≤ 1.3 ml/24 h) [13], we observed a dry rate of 52.9% for SUI, 32.4% for mixed and overall 39.2%. Not surprisingly, women with mixed incontinence had a much lower “dry” rate; however, their ICIQ scores were very significantly improved and their IIQ scores indicated a high degree of benefit. This improvement is likely attributable to facilitating greater effectiveness of anticholinergics to treat detrusor overactivity when both the stress incontinence and POP are adequately addressed by the continence dish.

The present study could be criticized because only about one third of participants had symptoms of pure stress incontinence. However, patients were recruited from a regional referral centre that covers a radius of 250 km, with waiting times of 6 months for a new appointment. Hence, many of the simple stress incontinence complaints had already been resolved by local pelvic floor physiotherapists (who are quite well staffed across the region). Also, it may have been preferable to recommend that all patients with mixed incontinence use anticholinergic medication before evaluating efficacy, but we preferred to evaluate the “real-life” scenario. To some extent, this desire to mirror “real life” may have introduced bias to the study, as only patients who wished to use the device were included in the audit. Ideally, a validated satisfaction score should have been used. However, three questions on our score had almost identical content to the three questions of the validated score published by Burgio et al. in 2006 [20], except that we included the word “continence dish” instead of “this program” in one question.

It is interesting to note that the continence dish did not provide full continence to all women with pure SUI. Previous authors have shown that levator ani muscles provide dynamic support to the urethra through their connection to the endopelvic facia of the anterior vaginal wall [21]. Biopsies of the levator ani muscles obtained during pelvic reconstruction surgery have demonstrated fibrosis in some women with pure stress incontinence, and these women are more likely to have recurrence after surgery [22]. Evaluation of women interested in using a continence dish by three-dimensional ultrasound of the levator ani may provide useful predictive data.

## Conclusion

In conclusion, the continence dish appears quite well tolerated once initial fitting is satisfactory. This device offers an alternative to other vaginal pessaries (Introl 65% cure on pad test of urodynamically proven stress incontinence and Contiform 54% cure on pad test of stress incontinence symptoms) or surgery for stress incontinence. Now that this audit has provided quantitative data regarding objective efficacy, a randomized trial of these devices would be feasible and highly informative. With increasing concerns over the use of polypropylene mesh for mid-urethral slings, the present data should be made available to women with both pure stress and mixed incontinence.

## References

[1] Holst K, Wilson PD (1988). The prevalence of female urinary incontinence and reasons for not seeking treatment. NZ Med J.

[2] Biswas NC (1988). A silastic vaginal device for the treatment of stress urinary incontinence. Neurourol Urodyn.

[3] Moore KH, Simons A, Dowell C, Bryant C, Prashar S (1999). Efficacy and user acceptability of the urethral occlusive device in women with urinary incontinence. J Urol.

[4] Hahn I, Milson I (1996). Treatment of female stress urinary incontinence with a new anatomically shaped vaginal device (Conveen continence guard). Br J Urol.

[5] Moore KH, Foote A, Burton G, King J (1999). An open study of the bladder neck support prosthesis in genuine stress incontinence. Br J Obstet Gynecol.

[6] Morris AR, Moore KH (2003). The contiform device. Int Urogynecol J.

[7] Hanson LA, Schulz JA, Flood CG, Cooley B, Tam F (2006). Vaginal pessaries in managing women with pelvic organ prolapse and urinary incontinence: patient characteristics and factors contributing to success. Int Urogynecol J.

[8] Donnelly MJ, Powell-Morgan S, Olsen AL, Nygaard IE (2004). Vaginal pessaries for the management of stress and mixed urinary incontinence. Int Urogynecol J.

[9] Richter HE, Burgio KL, Brubaker L (2010). Continence pessary compared with behavioural therapy or combined therapy for stress incontinence: a randomized controlled trial. Obstet Gynecol.

[10] Nager CW, Richter HE, Nygaard I (2009). Incontinence pessaries: size, POP-Q measures and successful fitting. Int Urogynaecol J.

[11] Karmakar D, Mostafa A, Abdel-Fattah M (2017). A new validated score for detecting patient-reported success on postoperative ICIQ-SF: a novel two-stage analysis from two large RCT cohorts. Int Urogynaecol J.

[12] Versi E, Orrego G, Hardy E, Seddon G, Smith P, Anand D (1996). Evaluation of the home pad test in the investigation of female urinary incontinence. Br J Obstet Gynaecol.

[13] O’Sullivan R, Karantanis E, Stevermuer TL, Allen W, Moore KH (2004). Definition of mild, moderate and severe incontinence on the 24-hour pad test. Br J Obstet Gynaecol.

[14] Uebersax JS, Wyman JF, Shumaker SA, McClish DK, Fantl JA (1995). Short forms to assess life quality and symptom distress for urinary incontinence in women: the incontinence impact questionnaire and the urogenital distress inventory. Neurourol Urodyn.

[15] Corcos J, Behlouli H, Beaulieu S (2002). Identifying cut-off scores with neural networks for interpretation of the incontinence impact questionnaire. Neurourol Urodyn.

[16] Robert M, Mainprize TC (2002). Long-term assessment of the incontinence ring pessary for the treatment of stress incontinence. Int Urogynaecol J.

[17] Komesu YM, Ketai LH, Rogers RG, Eberhardt SC, Pohl J (2008). Restoration of continence by pessaries: magnetic resonance imaging assessment of mechanism of action. Am J Obstet Gynecol.

[18] Noblett KL, McKinney A, Lane FL (2008). Effects of the incontinence dish pessary on urethral support and urodynamic parameters. Am J Obstet Gynecol.

[19] Ulmsten U, Johnson P, Rezapour M (1999). A three year follow up of tension free vaginal tape for surgical treatment if female stress urinary incontinence. Br J Obstet & Gynaecol.

[20] Burgio KL, Goode PS, Richter HE, Locher JL, Roth DE (2006). Global ratings of patient satisfaction and perception of improvement with treatment for urinary incontinence: validation of three global patient ratings. Neurourol Urodyn.

[21] DeLancey JO (1997). The pathophysiology of stress urinary incontinence in women and its implications for surgical treatment. World J Urol.

[22] Hanzal E, Berger E, Koelbl H (1993). Levator ani muscle morphology and recurrent genuine stress incontinence. Obstet Gynecol.

